# Oxidative Stress in Heat Stress Nephropathy: Crosstalk

**DOI:** 10.1155/omcl/1708417

**Published:** 2026-07-23

**Authors:** Justice Afrifa, Eric Gyamerah Ofori, Evans Duah, Ibrahim W. Naveh-fio, Gabriel Pezahso Kotam, Yeboah Kwaku Opoku, Addae Boateng Adu-Gyamfi, Helena Acquah, Enoch Essiam, Samuel Essien-Baidoo, Kwame Kumi Asare, Richard K. D. Ephraim

**Affiliations:** ^1^ Department of Medical Laboratory Science, School of Allied Health Sciences, University of Cape Coast, Cape Coast, Ghana, ucc.edu.gh; ^2^ Department of Biology Education, Faculty of Science Education, University of Education, Winneba, Ghana, uew.edu.gh; ^3^ School of Health Systems and Public Health, Faculty of Health, University of Pretoria, Pretoria, South Africa, up.ac.za; ^4^ Department of Population and Health, University of Cape Coast, Cape Coast, Ghana, ucc.edu.gh; ^5^ School of Allied Health Sciences, University of Health and Allied Sciences, Ho, Ghana, uhas.edu.gh; ^6^ Infectious and Non-Communicable Diseases, Biomedical and Clinical Research Centre, University of Cape Coast, Cape Coast, Ghana, ucc.edu.gh

## Abstract

Heat stress (HS) is a growing public health concern as rising global temperatures and occupational hazards expose a significant portion of the world’s population to extreme heat. The kidneys are particularly vulnerable to heat‐induced injuries, with oxidative stress (OS) playing a central role in their pathogenesis. The process involves heat‐induced mitochondrial dysfunction and stimulation of inflammatory pathways. This leads to the production of excessive reactive oxygen species (ROS) that eventually overwhelm the kidney’s natural antioxidant defenses. The resultant OS directly damages lipids, proteins, and DNA in renal tubular cells, a condition that is worsened by systemic inflammation and renal hypoxia. Although cells initially activate protective mechanisms involving heat shock proteins (HSPs) to preserve mitochondrial integrity, prolonged heat exposure undermines these defenses, increasing stress and triggering apoptotic pathways. This article focuses on the impact of HS on kidney health and the role of OS in the pathogenesis of heat‐induced kidney injuries. Key markers of heat‐induced OS, such as lipid peroxidation products (e.g., malondialdehyde [MDA], F_2_‐isoprostanes, 4‐hydroxynonenal [4‐HNE], and 8‐hydroxy‐2′‐deoxyguanosine [8‐OHdG]) and their effects on hematological indices and inflammatory response are highlighted. Ultimately, clarifying these specific mechanisms is essential to guide the development of early diagnostic tools and targeted treatments. This will help to reduce kidney damage, especially in vulnerable populations. We suggest that strategies such as adequate hydration, educating vulnerable groups on signs of heat strain, wearing light clothing, and heat acclimatization can mitigate the adverse effects of the rising global temperatures on kidney health.

## 1. Introduction

About 40% of the world’s population resides in climate areas with normal daytime temperatures exceeding 30°C [1]. In healthy person, the body has efficient heat regulatory mechanisms, which adapt to temperature elevations up to a particular threshold [2]. However, extreme heat events such as heat waves can result in a more severe condition known as heat stroke, characterized by high body temperatures exceeding 40°C and neurological symptoms [3]. Heat stress (HS) thus ensues when the internally generated heat (metabolic heat) and heat from the environment overwhelm the body’s ability to dissipate heat [1]. Typically, HS results in the induction of clinical syndromes including heat stroke, heat exhaustion, heat syncope, and heat cramps [1, 2, 4].

HS has been associated with many notable health conditions, among which is causing damage to the kidneys independent of traditional risk factors such as diabetes and hypertension [5]. This condition, known as HS nephropathy (HSN), is marked by a gradual decline in kidney function, damage to renal tubules, and fibrosis in the interstitial areas [6]. Although there is increasing awareness of this condition, the specific molecular processes that lead to kidney damage due to HS are still not fully understood. Central to the mechanism of heat‐induced kidney injury (KI) is oxidative stress (OS) [7]. OS occurs when there is an imbalance between the production of reactive oxygen species (ROS) and the body’s ability to neutralize them [4, 8]. This imbalance causes ROS to attack essential cellular macromolecules indiscriminately, leading to DNA damage that compromises genomic integrity, protein oxidation that modifies enzyme activity, and lipid peroxidation of cell membranes [9, 10]. The body’s main defense against this attack consists of nonenzymatic compounds including glutathione (GSH), vitamins C and E, and uric acid, as well as enzymatic antioxidants like GSH peroxidase (GPx), catalase (CAT), and superoxide dismutase (SOD) [11, 12].

The kidney is highly susceptible to oxidative damage because of its high metabolic activity and extensive blood supply, which require continuous mitochondrial function for tubular reabsorption. In particular, the renal proximal tubules have a high concentration of mitochondria to facilitate active transport, making them both a significant source and a target for mitochondrial ROS [13]. Renal tubular epithelial cells depend significantly on mitochondrial oxidative phosphorylation to maintain energy‐intensive transport processes. Because sodium reabsorption is an ATP‐dependent mechanism, attempts to increase fluid conservation during hyperthermia and dehydration worsen the reduced oxygen delivery [7, 13]. This results in the depletion of ATP in the renal cortex, leading to the induction of ROS.

During heat‐stress moments, there is an increase in mitochondrial electron leakage along with the activation of enzymatic ROS‐producing systems such as NADPH oxidase and xanthine oxidase [14]. This causes elevated production of ROS such as superoxide anions (O^2−^), hydrogen peroxide (H_2_O_2_), and hydroxyl radicals (OH^−^) [7]. ROS accumulation triggers the release of inflammatory cytokines, activates redox‐sensitive transcription elements (such as nuclear factor‐kappa B [NF‐κB]), and obstructs renal microcirculation and endothelial dysfunction [14]. The ensuing inflammatory position attracts neutrophils and macrophages, which produce even more ROS. [14] This cycle gradually impairs tubular structure and function and promotes interstitial inflammation and fibrotic remodeling, which are hallmarks of HSN.

Additionally, OS disrupts the Nrf2 (nuclear factor erythroid 2‐related factor 2) pathway, which is the main regulator of the antioxidant response in cells. Nrf2 (released by its inhibitory partner, Kelch‐like ECH‐associated protein 1 [Keap1]) typically enters the nucleus to promote the production of genes that provide protection. However, persistent OS and inflammation in the backdrop of HSN hinder Nrf2 activation, resulting in a harmful cycle where the kidneys are unable to lessen ROS damage [14, 15]. As a result, hemodynamic instability, the production of mitochondrial ROS, the induction of inflammation, and compromised antioxidant defenses all play a multifaceted role in HSN. Physiological responses to HS vary among individuals and are influenced by a number of factors. Vulnerable populations, including the elderly, young children, individuals with chronic illnesses, and those engaged in strenuous physical activities, are at higher risk for heat‐related illnesses [16–18]. These groups may have impaired thermoregulatory responses or an increased baseline level of physiological stress, making them less able to cope with high temperatures.

Studies have demonstrated that extreme temperatures, which frequently coexist with dehydration, are the main drivers of KI among individuals in HS [1, 19, 20]. Crowe et al. [20] observed heat‐related symptoms such as cramping muscles, weakness, nausea, vomiting, tachycardia, hyperventilation, ataxia, etc.., in more than 50% of sugarcane workers. HS induces a prerenal state with vasoconstriction of the kidney tubules and a decline in glomerular filtration rate (GFR) if volume depletion is severe [21]. Despite being reversible, this decline can be a risk factor for chronic kidney disease (CKD) [22].

Establishing possible treatment targets and elucidating the pathophysiology of HSN therefore depend on an understanding of these OS‐mediated pathways. Thus, the impact of HS on renal health is discussed in this article, with an emphasis on the role of OS in the pathophysiology of heat‐induced kidney problems. By examining the mechanisms through which HS induces oxidative damage, we highlight the connections between environmental heat exposure, OS, and kidney function. Additionally, this review discusses the implications of these findings for public health and suggests strategies to mitigate the adverse effects of rising global temperatures on kidney health.

## 2. HS and Markers of HS‐Induced OS

High temperatures can induce an imbalance between the generation of ROS and the antioxidant defense mechanisms within cells and tissues, leading to OS [23, 24]. For example, superoxide radicals are produced when there is a mitochondrial electron leak and the electron transport chain is compromised. DNA, proteins, and lipids within cells can all sustain damage from the ROS produced. Under HS, the kidneys suffer the diversion of blood from the renal cortex, resulting in a hypoxic environment that stops mitochondrial electron transport and releases superoxide radicals [14]. Importantly, OS initiates the epithelial‐to‐mesenchymal transition (EMT) and NLRP3 inflammasome, thereby linking acute oxidative injury to chronic inflammation and renal fibrosis (6, 11].

### 2.1. Heat‐Stress‐Induced Lipid Peroxidation

The body’s reaction to excessive heat exposure includes dysregulation in the levels of markers of OS such as malondialdehyde (MDA) and thiobarbituric acid‐reactive species (TBARS) [25]. These two are the primary products of the peroxidation of polyunsaturated fatty acids (PUFAs) [26]. However, recent studies indicate that TBARS is not reliable because most of the TBA‐reactive material in human body fluids is reported not to be related to lipid peroxidation [11]. Meanwhile, other investigations have shown increased amounts of protein carbonyls and lipid peroxidation products, such as isoprostanes, hydrocarbons, and aldehydes, in serum, plasma, and tissues as potent indicators of OS [11, 27]. MDA concentrations have been reported to be at higher levels in renal patients with decreased antioxidant activity. The rise in OS markers appears to be linked to an enhanced inflammatory condition likely stemming from an activated state of immune cells [28]. Research indicates that blood exiting the kidney graft contains a high concentration of CD4+ lymphocytes (LYMs) and CXCR3 monocytes, which suggests ongoing inflammatory processes [11, 29]. Additionally, OS and lipid peroxidation seem to be deeply rooted in various age‐related pathological and dysfunctional conditions.

According to research done on broilers, acute HS increased MDA levels significantly more than chronic HS [30–32]. This is usually because the intensity and length of HS affect the activity of related enzymes like GSH, CAT, and SOD among others. In humans, studies have shown significant OS‐related increased MDA levels in metabolic syndrome patients [33], patients infected with the influenza H1N1 virus [34], and during paraspinal muscle damage [35]. The levels of the enzymes were elevated in acute hypoxic situations to shield cells from excessive superoxide production. Despite this, other mediating variables such as the anticipated variations in the degree of OS adaptation in different participants, the attributes of the afflicted tissue, and the particular organism implicated have an impact on the overall outcome [36].

### 2.2. HS and Hematological Indices

The wet‐bulb globe temperature (WBGT) index and HS are correlated in earlier research [37, 38]. The WBGT index has been demonstrated to be significantly correlated with hematological parameters, including mean cell volume (MCV), red blood cells (RBCs), white blood cells (WBCs), and LYMs. This index is used as a predictor variable for MDA and nitric oxide (NO) levels [36]. A study in humans has shown that long‐term heat exposure among foundry workers resulted in elevated serum osmolality and decreased MCV and WBC in the exposed group [36]. Choi and Pai [39] observed a decrease in MCHC and an increase in HCT, MCV, platelet levels, RDW, and eosinophils in a study involving brief exposure to heat. Among bakers in the city of Shahroud in Iran, a study conducted showed that concentrations of MDA and NO were significantly higher during heat‐stress exposure. These were identified as risk factors for abnormal WBC, RBC, LYM, and MCV [36].

HS induces hematological changes (such as in MCV, RBC, and WBC) that lead to increased blood viscosity, reduced microcirculatory perfusion, and heightened OS. These are associated with inflammation and are capable of adversely affecting the renal function. The renal tubular cells initially respond with defense mechanisms involving the unfolded protein response (PERK and IRE1) and heat shock proteins (HSPs) mediated by HSF1 [40]. However, when stress is prolonged, these responses shift towards apoptosis due to mitochondrial dysfunction, stress in the endoplasmic reticulum (ER), and activation of CHOP [40]. Together, these cellular and systemic processes contribute to tubular injury and oxidative damage in HSN.

### 2.3. HSP Expression as a Marker of HS‐Induced OS

In both prokaryotic and eukaryotic animals, HSPs are widely distributed and conserved protein families that preserve cellular proteostasis and shield cells from external stimuli [41]. There is growing evidence that molecular chaperones, such as HSPs, actively contribute to cytoprotection and other cellular functions [42]. There are numerous HSP families performing different functions and classified based on their molecular weights. These range from large‐molecular‐weight HSPs, such as HSP90, HSP70, and HSP60, to smaller‐molecular‐weight HSPs like HSP40 [41]. However, the main inducible HSP during HS is the HSP70 subfamily of genes. In healthy cells, its expression is low at rest, but when HS occurs, it is noticeably elevated. HSPs expression is upregulated as a cellular defense mechanism against numerous stimuli, including glucose deprivation [43], hyperthermia [44], exercise [44], and OS [45, 46]. HSPs induced by HS have the potential to trigger a renal inflammatory response [47]. In some studies where HSP72 was overexpressed or preinduced, organ damage was prevented [48, 49].

According to Cong et al. [50], HSP72 can inhibit ROS caused by HS when puerarin, a traditional Chinese medicinal ingredient, is applied to bovine Sertoli cells. In animal studies, HSP72 expression was upregulated in proportion to the severity of heat stroke in most organs in rats and baboons [51]. Cells in which the expression of HSP70 is constitutively elevated demonstrate reduced breakdown of the common death substrate protein poly (ADP‐ribose) polymerase (PARP), which is linked to the inhibition of heat‐induced apoptosis [49]. Thus, HSP70 has the ability to prevent apoptosis. Among human subjects, varying results have been reported. At an ambient air temperature greater than 39°C, serum HSP70 levels were elevated above normal. A study by Butler‐Dawson et al. [52] found increased levels of HSP70 among sugarcane workers in relation to heat exposure. In contrast, heatstroke subjects had no increase in the levels of serum HSP70 [53]. Other studies have shown that exercise‐induced HS promoted the induction of HSP72 expression and its subsequent release into circulation [54].

### 2.4. HS‐Induced Elevated Proinflammatory Response

HS can trigger proinflammatory cytokines such as tumor necrosis factor‐alpha (TNF‐α) and interleukin‐6 (IL‐6) and activate inflammatory pathways. These elements encourage the generation of ROS in immune cells, which in turn causes OS. ROS serve as vital secondary messengers that activate redox‐sensitive transcription factors, particularly NF‐κB and the NLRP3 inflammasome [55, 56]. The activation of these pathways promotes the release of proinflammatory cytokines such as TNF‐α, IL‐1β, and IL‐6, creating a harmful cycle where inflammation worsens OS and vice versa [57]. In the kidney, this interaction leads to the apoptosis and pyroptosis of renal tubular epithelial cells, dysfunction of endothelial cells, and the advancement of heat‐stress‐induced nephropathy.

Autophagy and Nrf2 activation are two antioxidant responses that are triggered by heat‐stress‐induced apoptosis [58]. It has been noted that in certain organs, such as the testis and kidneys, alterations in autophagy dynamics are essential regulators of the protective function of the Nrf2 signaling pathway. The testis is protected against the harmful effects of HS by lowering MDA levels and fostering an antioxidant state [58–60]. In the kidneys, Nrf2 is viewed not merely as an antioxidant but is acknowledged for its various effects, which can be either damaging or beneficial, depending on the specific conditions and background [61].

### 2.5. Loss of Calcium Homeostasis

The kidney is crucial in keeping stable the body’s calcium levels. The nephron reabsorbs around 98% of the calcium that is filtered, and this process is carefully controlled to guarantee calcium equilibrium [62]. HS has the potential to disrupt Ca^2+^ homeostasis, which can activate calcium‐dependent enzymes and increase the production of ROS. Numerous studies have found that the loss of Ca^2+^ homeostasis is a common cause of free radical generation in skeletal muscle [63, 64]. Through a reduction in the sarcoplasmic reticulum Ca^2+^ ATPase function, high temperatures may be a contributing factor to the loss of Ca^2+^ homeostasis. Elevated free Ca^2+^ levels in a cell can lower the potential of the mitochondrial membrane and cause electron leakage, mostly in complexes I and III, which will ultimately result in more ROS being produced [65, 66].

The largest organelle in eukaryotic cells, the ER, is where most intracellular protein synthesis, posttranslational modifications, protein folding and transport, calcium ion storage and regulation, as well as signal transduction take place. According to Westrate et al. [67], the ER interacts with other organelles in response to both internal and external stress. For example, the ER and mitochondrial interactions help to maintain cellular Ca^2+^ homeostasis [68]. Stress impairs the ER’s capacity to process or transport proteins and control the uptake and release of Ca^2+^. The buildup of unfolded and misfolded proteins in the ER lumen as a result of this dysfunction disrupts calcium homeostasis [69, 70]. The disruption is a critical factor that connects OS to damage in renal cells, making it a possible target for treatment [71].

Calcium overload induced by HS, along with increased ROS, activates the opening of the mitochondrial permeability transition pore (mPTP), resulting in a loss of membrane potential and apoptosis mediated by cytochrome c [72–74]. The degree of cellular damage influences the outcome. Temporary mPTP opening promotes apoptosis, while prolonged calcium influx leads to ATP depletion and necrosis, which exacerbates inflammation and kidney damage in HSN [75].

## 3. HS, OS, and Kidney Damage

The mechanism by which HS induces nephropathy is not fully understood. However, it has been attributed to the excessive amount of excess fluid and electrolyte losses from the body due to frequent heat exposure [76]. Dehydration leads to a decrease in blood pressure. Low blood pressure leads to ischemia in the kidney and accumulation of ions in the tubules, with its attendant decline in GFR [76]. Over time, the accumulated ions clog the tubules and lead to the loss of the nephrons, glomerulosclerosis, glomerular hypertrophy, signs of chronic glomerular ischemia, and mild‐to‐moderate chronic tubulointerstitial damage [76, 77]. Prolonged exposure to heat can also trigger exertional rhabdomyolysis, a pathological condition marked by severe damage to the skeletal muscle tissue [78]. Exertional rhabdomyolysis leads to the release of various catabolic end products, including myoglobin, creatine kinase, electrolytes, troponin, aldolase, and lactate dehydrogenase, into circulation. In particular, the excessive precipitation of myoglobin in the renal tubules, often manifested as myoglobinuria, can obstruct the tubules and impair urine output [79]. This obstruction may restrict blood flow to the renal tubular cells, leading to ischemia and subsequent tubular necrosis. Extreme temperatures negatively affect the renal tubular epithelial cells, especially in the proximal tubules. This injury arises when mitochondria damaged by heat interfere with ATP production, compromising cell survival.

Several studies have reported kidney dysfunction among workers exposed to heat. In mice, it was observed that when the core body temperature was increased, KI in HS worsened [13]. Among humans, studies have shown that participating in a muscle‐damaging exercise before running in the heat may result in an elevated inflammatory state and kidney damage. There is also evidence to show that among hospitalized patients in the US, approximately one‐third of heat stroke patients developed AKI [80]. While there have been documented cases of heat stroke‐induced AKI and CKD [80, 81], early detection of myoglobinuria can guide prompt management decisions.

ROS can also be generated from oxidative phosphorylation via electron leakage from the mitochondrial electron transport chain. This is especially so when uncoupling proteins such as UCP1 and mitochondrial transporter proteins like adenine nucleotide translocase (ANT) are disrupted [82, 83]. ROS generation is also increased when mitochondrial respiratory complexes, particularly I, III, and IV, are suppressed [82, 83]. Elevated temperatures can also induce oxidative damage to mitochondrial phospholipids, leading to lipid peroxidation and suppression of electron transfer in the electron transport chain [84].

**Figure 1:**
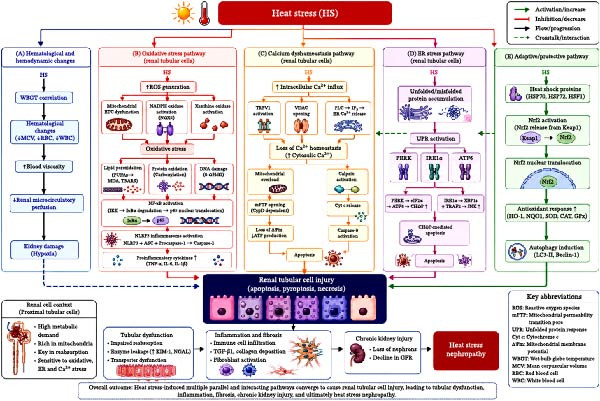
A framework exploring the interconnected pathways of heat stress, oxidative stress, and kidney injury.

OS and kidney pathology are intricately linked, with each capable of exacerbating the other through a feedback loop (Figure 1). This interaction creates a vicious cycle of cellular damage and organ dysfunction. Elevated levels of ROS in circulation, resulting from cellular mitochondrial damage due to HS, can exacerbate the effects of hypoperfusion and hypoxia on the renal medulla, leading to AKI and its progression [85]. Additionally, ROS can induce vasoconstriction and increase vascular resistance in the renal tubules, which are critical factors in kidney dysfunction [86]. These renal tubular cells, abundant in mitochondria, are particularly vulnerable to OS [87, 88] as they require significant energy for solute and water reabsorption into circulation. Ischemic injury in these cells may result in the release of hydroxyl radicals, peroxynitrite, and hypochlorous acid ROS while depleting antioxidant enzymes such as SOD, CAT, and GSH reductase [89].

Similarly, a vicious cycle exists between ROS and KI (Figure 1). ROS plays a role in both the onset and progression of KI, while KI, in return, heightens ROS production and OS, exacerbating kidney damage and dysfunction [90]. Increased systemic OS is generally associated with kidney damage. For instance, elevated systemic ROS levels contribute to the damage of renal tubular cellular structures, including DNA, mitochondria, membrane phospholipids, and proteins, ultimately leading to kidney dysfunction [91]. A summary of HS sources, related subjects, markers, and the response to such HS is provided in Table 1.

**Table 1:** Summary of heat stress sources, markers, and response to their respective responses.

Reference	Year	Subject	Heat stress source	Marker	Response	
[44]	2001	Human athletes	Environmental heat and exercise	HSP72	Cytoprotection	
[46]	2000	Rats	Acute exercise	HSP72	Cytoprotection	
[50]	2017	Bovine Sertoli cells	Laboratory heat	HSP72	Cytoprotection	
[51]	2010	Baboons	Environmental heat	HSP72	Cytoprotection	
[48]	2006	Transgenic mice	Laboratory heat	HSP72	Cytoprotection	
[53]	2006	Human subjects	Environmental heat	HSP70	Autoantibody/cytoprotection	
[52]	2024	Human subjects	Environmental heat	HSP70	Autoantibody/cytoprotection	
[92]	2010	Chicken	Controlled heat exposure	MDA	Lipid peroxidation	
[30]	2009	Chicken	Controlled heat exposure	MDA, 3HADH	Lipid peroxidation	
[93]	2013	Chicken	Controlled heat exposure	MDA	Lipid peroxidation	

## 4. Mitigating the Effects of Heat‐Stress‐Induced KI

HS can affect human health and cause damage to several organs, notably the kidneys and heart [5]. In events of increased temperature, there is increased loss of fluid through sweat and respiration, while loss through urine and feces (sensible fluid loss) is decreased, leading to a hypovolemic state. Hypoperfusion of the kidneys ensues as the remaining blood is redistributed to the muscle and skin to dissipate heat [94]. Dehydration induces a prerenal state with vasoconstriction of the kidney tubules and a decline in GFR if dehydration is severe [21]. Adequate fluid intake, weight measurement to ascertain dehydration, training workers to recognize signs of HS, and heat acclimation are ways that have been postulated to reduce the effects of HS on the kidneys.

### 4.1. Adequate Fluid Intake

Regular and adequate fluid intake may be the most important intervention to help reduce the effects of HS on the kidneys [1], although it may not necessarily eliminate their effects [94, 95]. Adequate water intake restores fluid balance and promotes optimal kidney function by supporting sufficient blood flow to the kidneys, helping prevent complications and maintain overall health. Again, dehydration stimulates the release of ADH, which conserves water by reabsorbing water from urine in the collecting ducts of the kidneys. This makes urine supersaturated with substances like calcium, oxalate, and uric acid, thereby increasing the risk of renal calculi formation [21, 96]. Highly motivated individuals, mostly those with low socioeconomic status, who pay per output and have the risk of losing employment may exert themselves beyond safe limits, causing severe health consequences [97]. Due to these motivations, individuals, although well aware of the importance of hydration, may overlook it. In these individuals, weight measurement, urine appearance, and the degree of thirst should be used as indicators for water intake. Adequate water mitigates this effect by diluting urine and preventing the supersaturation of salts. However, in a bid to rehydrate, individuals should avoid soft drinks, as these have the potential to worsen dehydration and exacerbate the effects of HS on the kidneys [98]. These beverages (energy drinks, soft drinks, etc.) often contain high levels of sugar and low water content, leading to increased fluid loss through the urine in an attempt to expel the excess sugar [99].

### 4.2. Body Weight Assessment and Urine Color

Water makes up ~60% of total body weight in adults [100]. When the body loses water due to heat exposure, it results in a decrease in the total body weight. This weight loss is directly proportional to the amount of water lost due to the significant contribution of water to body weight [101]. Constant monitoring of body weight should thus be implemented in workplaces that are exposed to high temperatures [101]. Urine color is a reliable indicator of hydration status [102, 103]. Urine color is very responsive to changes in fluid balance. In well‐hydrated individuals, urine is straw‐colored but dark yellow or amber in dehydrated individuals [102, 104]. Recognizing these changes can prompt individuals to take proactive steps to maintain proper hydration levels. Dehydration again activates thirst centers in a bid to replenish lost fluid. These are signs that can easily be observed and can inform individuals about their hydration status.

### 4.3. Wearing of Light Clothes

Wearing light clothing allows for better air circulation and facilitates the evaporation of sweat, aiding in the maintenance of body temperature [105]. This optimizes the body’s natural cooling mechanisms and supports homeostasis in challenging environmental conditions, reducing the effects of HS. The evaporation of sweat is substantially hindered when clothing is thick and layered. Because of this, working with personal protective clothing (PPC), especially in warm climates and among factory workers, implies significant physiological strain and may render workers easily exhausted [106]. McLellan and Selkirk [107] concluded in 2004 that replacing the long pants of firefighters with shorts reduced the HS associated with the protective ensemble during light exercises. This is because the shorts decrease the thermal resistance. Individuals engaged in strenuous exercise and exposed to extreme temperature conditions should thus opt for shorts and lighter clothes to limit the effects of HS.

### 4.4. Heat Acclimation

Heat acclimation has been proposed as a strategy that can improve kidney function during heat exposure and minimize the risk of heat‐induced KI [17, 108]. Repeated heat exposure increases whole‐body temperature and stimulates profuse sweating, which primes the system by increasing blood volume, decreasing core and skin temperatures, and inducing important molecular adaptations that stimulate physiological heat acclimation [109]. Heat acclimation provides thermal tolerance, which is associated with an elevation of HSPs [94, 110]. HSPs are crucial for kidney survival, and their induction through heat acclimation represents a protective mechanism against heat‐induced KI. Earlier studies revealed that doing exercise in the heat for 4 days in a row did not change kidney perfusion in young, healthy adults. The 4‐day heat acclimation protocol was sufficient to stimulate plasma volume expansion, suggesting evidence of heat adaptation [111]. We propose that recruits or individuals starting work in high‐temperature environments consider transient exposure to the work environment for about a week before beginning active work.

## 5. Conclusion and Future Perspective

Evidence points to the fact that excessive heat exposure contributes to the production of ROS, which could overburden the body’s antioxidant defenses and trigger inflammation, endothelial dysfunction, and alterations in overall physiology.

Information on the extent of oxidative damage and the body’s heat‐stress adaptive responses can be obtained from the increase in levels of a number of valuable markers, including lipid peroxidation products, GPx, SOD, CAT, and protein carbonyls, among others. Although the body initially attempts to cope through reactions such as generating HSPs to protect kidney function, persistent thermal stress increases the likelihood of mitochondrial dysfunction, ER stress, and the onset of apoptosis. Furthermore, accurately diagnosing this form of injury typically calls for specific OS biomarkers like F_2_‐isoprostanes, 4‐hydroxynonenal (4‐HNE), 8‐hydroxy‐2′‐deoxyguanosine (8‐OHdG), and nitrotyrosine.

The current findings underscore that HSN is not solely attributed to temperature exposure but also appears to involve intricate interactions between body physiology and cellular stress responses. It is a complex condition associated with the interplay of OS, inflammation, calcium dysregulation, ER stress, cellular stress responses, and broader systemic changes.

To lessen this burden, a comprehensive approach involving lifestyle modifications, preventive measures, and novel therapeutic approaches is recommended. Workplace changes like planned rest periods and improved ventilation, as well as heat acclimatization, have all been associated with reductions in the incidence of heat‐related renal damage. At the molecular level, antioxidant therapies and anti‐inflammatory medications have demonstrated potential in preventing heat‐stress‐induced oxidative damage and inflammatory cascades.

Despite significant advances in understanding the role of OS in HSN, there are still significant mechanistic questions about pathway hierarchy, signaling crosstalk dynamics, and threshold impacts. Future research should concentrate on in vivo models and long‐term human studies to clarify these mechanisms. This could help with early detection and targeted therapies, such as the potential of using Nrf2 activators and mitochondrial stabilizers for kidney protection.

## Author Contributions

**Justice Afrifa**: writing – review and editing, writing – original draft, visualization, validation, conceptualization, literature search. **Eric Gyamerah Ofori**: writing – review and editing, writing – original draft, visualization, validation, conceptualization. **Evans Duah**: writing – review and editing, visualization, validation, writing – original draft. **Ibrahim W. Naveh-fio and Addae Boateng Adu-Gyamfi**: writing – review and editing. **Gabriel Pezahso Kotam**: writing – original draft, writing – review and editing. **Yeboah Kwaku Opoku**: figure editing, writing – review and editing. **Helena Acquah, Enoch Essiam, Samuel Essien-Baidoo, and Kwame Kumi Asare**: review and editing. **Richard K. D. Ephraim**: writing – review.

## Funding

No funding was received for this manuscript.

## Conflicts of Interest

The authors declare no conflicts of interest.

## Data Availability

Data sharing is not applicable to this article, as no datasets were generated or analyzed during the current study.
